# Association between Achilles tendon abnormalities and systemic atherosclerosis in patients undergoing percutaneous coronary intervention

**DOI:** 10.1016/j.ijcha.2026.101971

**Published:** 2026-07-14

**Authors:** Yushi Oyama, Yasushi Ueki, Kyuhachi Otagiri, Tadashi Itagaki, Junko Iguchi, Takuya Miyagi, Takahiro Sakai, Koki Fujimori, Daisuke Sunohara, Yuki Yamamoto, Hidetomo Nomi, Tamon Kato, Tatsuya Saigusa, Hiroshi Kitabayashi, Soichiro Ebisawa, Koichiro Kuwahara

**Affiliations:** a Department of Cardiovascular Medicine, Ina Central Hospital, Ina, Japan; b Department of Cardiovascular Medicine, Shinshu University School of Medicine, Matsumoto, Japan

**Keywords:** Achilles tendon, Achilles tendon xanthomas, Achilles tendon thickening, Atherosclerosis, Polyvascular disease, Ultrasound

## Abstract

**Background:**

Tendon xanthomas, manifested as Achilles tendon (AT) thickening (ATT) or structural abnormalities, share pathogenic mechanisms with atherosclerosis such as cumulative exposure to low-density lipoprotein cholesterol; however, their association with the severity of systemic atherosclerosis remains unclear. This study sought to investigate the association between AT abnormalities assessed by ultrasound and the prevalence and severity of polyvascular disease (PVD) in patients undergoing percutaneous coronary intervention (PCI).

**Methods:**

This cross-sectional analysis used baseline data from a prospective multicenter observational study (Achilles Study: UMIN000053786). AT thickness and structural abnormalities were evaluated using ultrasonography. ATT was defined as ≥6.0 mm in men and ≥ 5.5 mm in women. AT structural abnormalities were defined as the presence of calcification, abnormal layer structure, or localized hypoechogenicity.

**Results:**

Among 333 patients (median age 75 years; 83% male), ATT was observed in 109 patients (33%), and AT structural abnormalities were identified exclusively in patients with ATT (14%). The prevalence of PVD increased stepwise across patients with no AT abnormalities, those with ATT without structural abnormalities, and those with ATT with structural abnormalities (20% vs. 30% vs. 51%, *p* < 0.001). Maximum intima-media thickness of carotid artery and SYNTAX score were highest among patients with both ATT and structural abnormalities. These associations remained consistent in patients without familial hypercholesterolemia (FH).

**Conclusions:**

AT structural abnormalities assessed by ultrasound were significantly associated with the prevalence and severity of PVD in patients undergoing PCI. Assessment of AT abnormalities may serve as a useful marker for evaluating systemic atherosclerotic burden.

## Highlights

• AT structural abnormalities was observed in 14% of CAD patients undergoing PCI.
• Structural abnormalities were associated with the prevalence and severity of PVD.
• AT abnormalities may serve as a useful marker of systemic atherosclerotic burden.

## 1 Introduction

Achilles tendon (AT) thickening (ATT) is widely recognized as a manifestation of tendon xanthomas and constitutes one of the diagnostic criteria for familial hypercholesterolemia (FH) [1]. AT xanthoma (ATX) is considered to develop as a consequence of prolonged exposure to elevated low-density lipoprotein cholesterol (LDL-C) leading to cholesterol deposition within extravascular tissues [2]. Histologically, tendon xanthomas comprise form cells derived from monocyte, extracellular cholesterol, and connective tissue. Given the mechanistic overlap with atherosclerotic plaque formation, ATX may serve as a useful surrogate marker of the burden and progression of atherosclerotic cardiovascular disease (ASCVD).

Assessment of the AT is commonly performed using either ultrasonography or soft-tissue radiography [1]. However, radiography is limited in the accuracy to assess AT thickness due to its low image resolution. In contrast, ultrasonography can provide more accurate measurements of AT thickness with greater reproducibility in a non-invasive manner [3], [4]. In addition, while soft-tissue radiography can detect calcification, ultrasonography provides more comprehensive evaluation of structural abnormalities within the AT, including calcification, abnormal layer structure, and localized hypoechogenicity suggestive of lipid deposition [4], [5]. Previous studies have reported that AT structural abnormalities are associated with greater coronary artery disease (CAD) severity and worse clinical outcomes [5], [6]. However, no data are currently available regarding the relationship between AT structural abnormalities and systemic atherosclerotic burden. Therefore, we examined the relationship between AT abnormalities assessed by ultrasound and the prevalence and severity of polyvascular disease (PVD) in patients undergoing percutaneous coronary intervention (PCI).

## 2 Methods

### 2.1 Study population

Consecutive patients undergoing PCI and AT thickness assessment were enrolled into the Achilles Study, a prospective, multicenter, observational study (UMIN000053786). The current study is a cross-sectional analysis using baseline data from patients enrolled to the Achilles Study. For the current study, patients with non-atherosclerotic causes of acute coronary syndrome (ACS), a prior history of bilateral AT rupture, or a prior history of Achilles tendinitis were excluded. The current study was approved by the ethics committee of Ina Central Hospital (approval no. 23–1) and was performed in conformity with the Declaration of Helsinki. In the current study, smoking history included both current and former smokers. Plasma lipid levels were measured at hospital admission prior to PCI. PVD was defined as the presence of more than 2 coexisting atherosclerosis diseases including CAD, carotid artery disease or atherothrombotic brain infarction, and peripheral artery disease including lower extremity artery disease (LEAD), subclavian artery stenosis, and renal artery stenosis. Carotid artery disease was defined as the presence of ≥50% carotid artery stenosis, including asymptomatic carotid stenosis, or a history of carotid revascularization [7]. LEAD was defined as an ankle–brachial index ≤0.90 or a history of lower-extremity revascularization [7]. FH was diagnosed during PCI hospitalization, based on the 2022 guidelines of the Japan Atherosclerosis Society FH criteria [1].

### 2.2 Evaluation of the AT

**Fig. 1:**
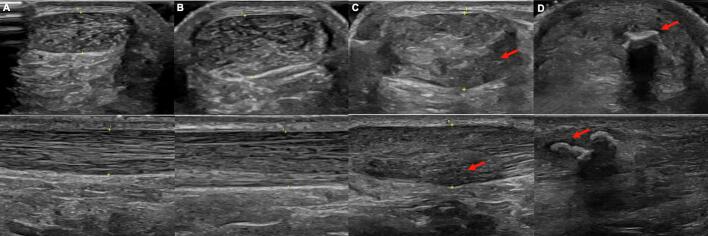
Representative ultrasonographic images of normal and abnormal Achilles tendons. The upper and lower panels represent short-axis and long-axis views, respectively. (A) Normal Achilles tendon, (B) Achilles tendon thickening without structural abnormalities, (C) Achilles tendon showing abnormal layer structure and localized hypoechogenicity (red arrow), (D) Achilles tendon with calcification (red arrow).

AT thickness was measured by ultrasound according to the guidelines by the Japan Atherosclerosis Society during PCI hospitalization [1]. In brief, the subject was placed in either kneeling or supine position on the bed. AT thickness was measured at the thickest point on both sides and the maximum thickness was used for the analysis. The ATT was defined as ≥6.0 mm in men and ≥ 5.5 mm in women. AT structural abnormalities were defined as the presence of calcification, abnormal layer structure, and localized hypoechogenicity (Fig. 1). The testing equipment and ultrasound probes comprised an Aplio i700 device (Canon Medical, Otawara, Japan), a high-frequency linear type probe with a central frequency of 18 MHz, and a LOGIQ E10 device (GE Healthcare Japan, Tokyo, Japan), and a high-frequency linear type probe with a central frequency of 15 MHz. The measurements were performed by expert sonographers who were blinded to the clinical data of the subjects. The inter-rater reliability of AT thickness measurements was assessed in 11 randomly-selected patients (5 at Shinshu University Hospital and 6 at Ina Central Hospital) using the intraclass correlation coefficient (ICC). The ICC for interobserver agreement was 0.99 (95% Confidence Interval [CI]: 0.96–1.00). In addition, the inter-rater reliability for the assessment of AT structural abnormalities was evaluated in 40 randomly selected patients (20 at Shinshu University Hospital and 20 at Ina Central Hospital) using the kappa coefficient. The kappa coefficient for interobserver agreement was 0.93 (95% CI: 0.80–1.00).

### 2.3 Evaluation of the carotid maximum intima-media thickness

Maximum intima-media thickness (max-IMT) of the common carotid artery was measured using the same equipment used for AT thickness assessment and an EPIQ CVx (Philips Medical Systems, Andover, MA, USA) with a high-frequency linear type probe with a central frequency of 12 MHz, in accordance with the guidelines of the Japanese Society of Ultrasound Medicine, during hospitalization for PCI [8]. The greater value of max-IMT between the right and left common carotid arteries was used for analysis [9], [10]. Carotid ultrasonography was performed in all patients enrolled to the current study; however, those with a history of carotid artery stenting or carotid endarterectomy were excluded from analyses related to carotid max-IMT.

### 2.4 Evaluation of the severity of CAD

CAD severity was assessed by the SYNergy between percutaneous coronary intervention with TAXus and cardiac surgery (SYNTAX) score using the SYNTAX score calculator (available at http://www.syntaxscore.com) based on invasive coronary angiography before PCI [11]. Two experienced interventional cardiologists who were blinded to the patient clinical data including AT thickness visually assessed all coronary segments ≥1.5 mm in diameter for detecting the presence of significant stenotic lesions defined as luminal diameter stenosis ≥50%. Patients with a history of coronary artery bypass grafting were excluded from analyses related to the SYNTAX score.

### 2.5 Statistical analysis

All statistical analyses were performed with R (The R Foundation for Statistical Computing, Vienna, Austria) and EZR (Saitama Medical Center, Jichi Medical University, Saitama, Japan). Because missing data were minimal for each variable, no imputation was performed, and analyses were conducted using available-case analysis. Continuous variables were summarized as median and interquartile ranges, and compared between the groups using the Kruskal-Wallis test. Binary and categorical variables were calculated as frequencies (percentages), and were compared with the chi-square test or Fischer's exact test. A value of *p* < 0.05 was accepted as statistically significant. A multivariate logistic regression models was performed to identify an independent determinant for the prevalence of PVD. AT status and clinically important variables including age, sex, hypertension (HT), diabetes mellitus (DM), dyslipidemia (DLP), smoking history, and chronic kidney disease (CKD) were entered into the model. A multiple linear regression analysis was also performed to identify an independent determinant for SYNTAX score.

## 3 Results

**Fig. 2:**
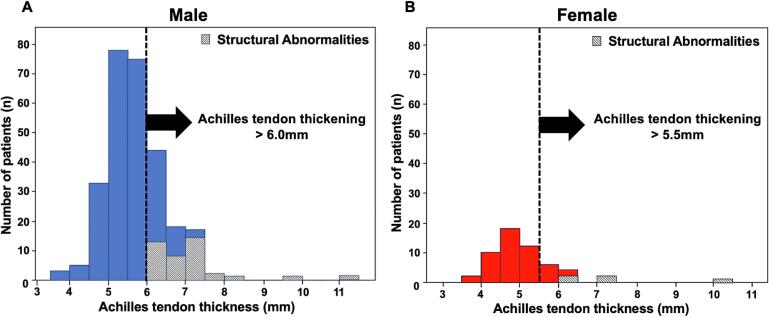
Distribution of Achilles tendon thickness and structural abnormality according to sex. (A) Male, (B) Female.

**Fig. 3:**
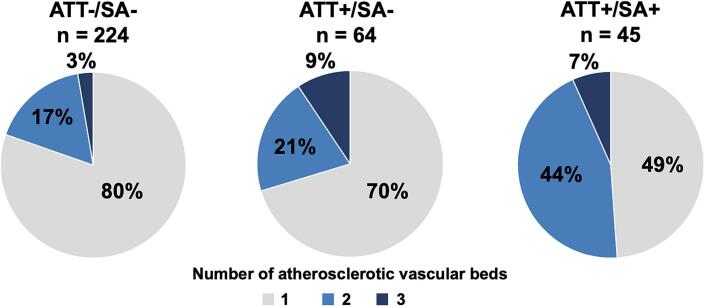
Number of atherosclerotic vascular beds according to Achilles tendon status. ATT: Achilles tendon thickening, SA: Structural abnormalities.

**Fig. 4:**
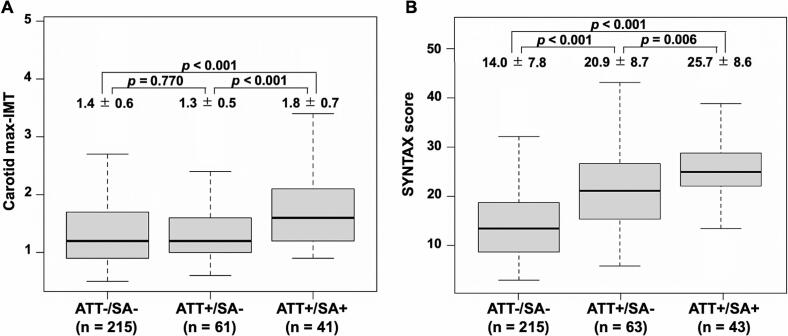
Carotid max-IMT and SYNTAX score according to Achilles tendon status. (A) Comparison of carotid max-IMT according to Achilles tendon status (*n* = 317). (B) Comparison of SYNTAX score according to Achilles tendon status (*n* = 321). Max-IMT: Maximum intima-media thickness, SYNTAX: SYNergy between percutaneous coronary intervention with TAXus and cardiac surgery, ATT: Achilles tendon thickening, SA: Structural abnormalities.

**Supplementary Fig. S1:**
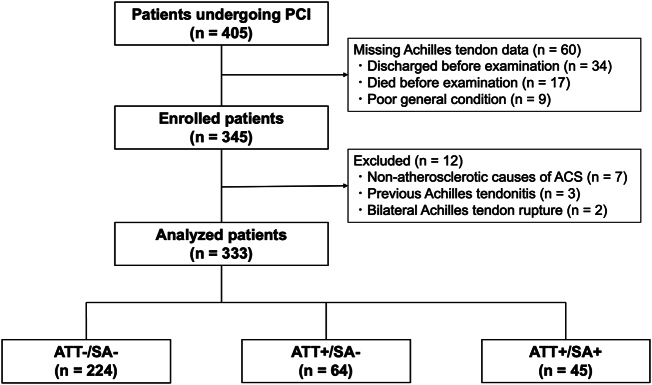
Study flow chart.

**Supplementary Fig. S2:**
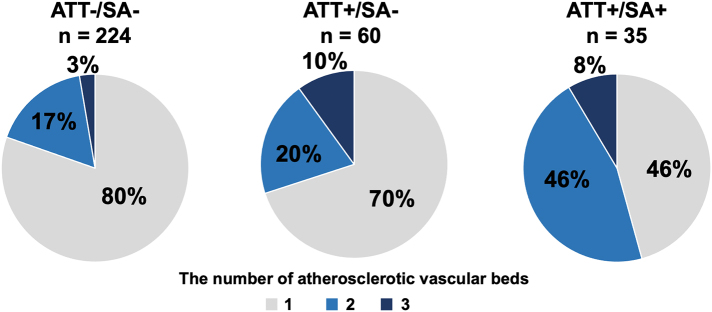
Number of atherosclerotic vascular beds according to Achilles tendon status in patients without familial hypercholesterolemia.

**Supplementary Fig. S3:**
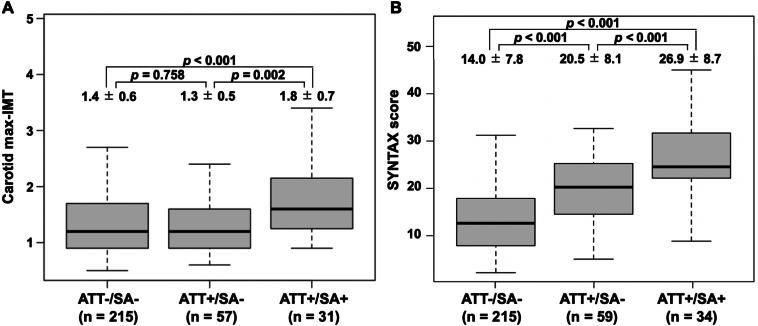
Association of Achilles tendon status with carotid max-IMT and SYNTAX score in patients witout familial hypercholesterolemia.

Of 345 patients enrolled into the Achilles study between May 2023 and April 2025, after excluding 12 patients (non-atherosclerotic causes of ACS: *n* = 7, previous Achilles tendonitis: *n* = 3, bilateral AT rupture: *n* = 2), 333 patients (median age 75 [66–81] years; 83% male) were analyzed for the current study (Supplementary Fig. 1). The distribution of AT thickness and structural abnormalities is shown in Fig. 2. ATT was observed in 109 patients (33%), and AT structural abnormalities were identified exclusively among patients with ATT in 45 cases (14%). Among patients with AT structural abnormalities, all exhibited abnormal layer structure and localized hypoechogenicity, whereas calcification was observed in 2 patients. Overall, PVD was present in 86 patients (26%), FH was identified in 14 patients (4%), and multivessel CAD was observed in 167 patients (50%). When patients were classified into three groups according to AT status (no abnormality [ATT-/SA-]: *n* = 224, ATT without structural abnormalities [ATT+/SA-]: *n* = 64, and ATT with structural abnormalities [ATT+/SA+]: *n* = 45), patients with ATT+/SA+ had a significantly higher prevalence of CKD including hemodialysis (HD), FH, and multivessel CAD (Table 1). In contrast, patients with ATT+/SA- were more often female and more likely to present with ACS. Furthermore, there was no significant difference in the prevalence of premature CAD (<55 years in men and < 65 years in women) among the three groups. These trends were consistently observed in the non-FH cohort (Supplementary Table 1). The prevalence of PVD was higher in patients with ATT+/SA+ compared with other groups (ATT-/SA-: 20%, ATT+/SA-: 30%, ATT+/SA+: 51%, *p* < 0.001). Notably, patients with two coexisting atherosclerotic diseases were most prevalent in the ATT+/SA+ group (17%, 21%, 44%, *p* < 0.001) (Fig. 3). Among 317 patients with available carotid max-IMT measurements, carotid max-IMT was significantly greater in the ATT+/SA+ group compared with the other groups (Fig. 4A). The SYNTAX score showed a stepwise increase across the three groups (Fig. 4B). A similar trend was observed among patients without FH (Supplementary Figs. 2 and 3).

**Table 1:** Baseline characteristics.

Variable	ATT-/SA- (*n* = 224)	ATT+/SA- (*n* = 64)	ATT+/SA+ (*n* = 45)	*p* value
Age (years)	75 [67–81]	76 [68–82]	72 [59–79]	0.071
Body mass index (kg/m^2^)	23.3 [21.6–25.3]	22.3 [20.5–24.9]	23.9 [22.0–26.0]	0.058
Male	194 (87%)	44 (69%)	40 (89%)	0.003
Hypertension	166 (74%)	47 (73%)	29 (64%)	0.412
Dyslipidemia	168 (75%)	43 (67%)	35 (78%)	0.380
Diabetes mellitus	91 (41%)	29 (45%)	21 (47%)	0.672
Smoking history	159 (71%)	35 (55%)	30 (67%)	0.051
Family history of CAD	31 (14%)	10 (16%)	9 (20%)	0.590
Renal failure (eGFR <60 ml/min/1.73m^2^)	111 (50%)	43 (67%)	30 (67%)	0.012
eGFR (mL/min/1.73m^2^)	60 [50–73]	55 [41–71]	54 [40–63]	0.018
Hemodialysis	8 (4%)	4 (6%)	7 (22%)	0.010
Previous PCI or CABG	46 (21%)	15 (23%)	12 (27%)	0.606
Polyvascular disease	44 (20%)	19 (30%)	23 (51%)	<0.001
Lower extremity artery disease	23 (10%)	10 (16%)	13 (29%)	0.006
ATBI or carotid artery disease	28 (13%)	15 (23%)	12 (27%)	0.015
Multivessel CAD	94 (42%)	39 (61%)	34 (76%)	<0.001
Familial hypercholesterolemia	0 (0%)	4 (6%)	10 (22%)	<0.001
Premature CAD	15 (7%)	6 (9%)	5 (11%)	0.427
ACS presentation	109 (49%)	41 (64%)	19 (42%)	0.044
Prior statin therapy	107 (48%)	28 (44%)	20 (44%)	0.830
Prior ezetimibe therapy	22 (10%)	2 (3%)	6 (13%)	0.117
Prior PCSK9-i therapy	0 (0%)	0 (0%)	0 (0%)	N/A
Total cholesterol (mg/dL)	176 [147–209]	186 [158–205]	179 [151–215]	0.752
LDL cholesterol (mg/dL)	98 [77–129]	107 [89–126]	109 [82–137]	0.427
Triglyceride (mg/dL)	116 [81–175]	119 [73–161]	119 [68–180]	0.814
HDL cholesterol (mg/dL)	49 [41–59]	49 [42–58]	47 [42–53]	0.579

Values are n (%) or median [interquartile range].

All continuous variables were analyzed using the nonparametric Kruskal-Wallis test.

ATT: Achilles tendon thickening, SA: Structural abnormalities, CAD: Coronary artery disease, eGFR: estimated glomerular filtration rate, PCI: Percutaneous coronary intervention, CABG: Coronary artery bypass grafting, ATBI: Atherothrombotic brain infarction, ACS: Acute coronary syndrome, PCSK9-i: Proprotein convertase subtilisin/kexin type 9 inhibitor, LDL: Low-density lipoprotein, HDL: High-density lipoprotein.

In a multivariate logistic regression analysis for the prevalence of PVD adjusted for age, sex, HT, DM, DLP, smoking status, and CKD, the ATT+/SA+ group was significantly associated with the prevalence of PVD (OR, 6.000; 95% CI, 2.820–12.800; *p* < 0.001) (Table 2). Similar results were also observed among patients without FH and those not receiving HD. (Supplementary Tables 2 and 3). Additionally, the prevalence of HD did not differ significantly between patients with and without PVD (10% vs. 4%, *p* = 0.054). Furthermore, AT structural abnormalities were independently associated with a higher SYNTAX score after adjustment for clinically relevant covariates in the multivariable linear regression analysis (Supplementary Table 4).

**Table 2:** Univariate and multivariate logistic regression analysis for prevalence of polyvascular disease.

Variable	Univariate analysis			Multivariate analysis		
	OR	95% CI	*p* value	OR	95% CI	*p* value
Achilles tendon Status						
ATT-/SA-	1.000 (reference)			1.000 (reference)		
ATT+/SA- (vs. ATT-/SA-)	1.730	0.920–3.240	0.088	1.890	0.959–3.730	0.066
ATT+/SA+ (vs. ATT-/SA-)	4.280	2.190–8.380	<0.001	6.000	2.820–12.800	<0.001
Age	1.030	1.000–1.050	0.025	1.040	1.010–1.070	0.005
Male	1.480	0.725–3.010	0.282	1.220	0.500–2.980	0.661
Hypertension	2.100	1.130–3.910	0.019	2.190	1.110–4.330	0.024
Diabetes mellitus	1.840	1.120–3.020	0.016	1.700	0.995–2.890	0.052
Dyslipidemia	0.958	0.549–1.670	0.880	0.943	0.510–1.740	0.850
Smoking	1.460	0.849–2.520	0.171	1.920	0.948–3.870	0.070
Chronic kidney disease	1.740	1.040–2.890	0.034	0.994	0.553–1.790	0.985

OR: Odds ratio, CI: Confidence Interval, ATT: Achilles tendon thickening, SA: Structural abnormalities.

## 4 Discussion

This study is the first to demonstrate that ATT and structural abnormalities assessed by ultrasonography were associated with systemic atherosclerotic burden in patients undergoing PCI. Structural abnormalities were observed exclusively in patients with ATT and demonstrated incremental significance, correlating with a higher prevalence and greater severity of PVD. Similar trends were observed in the non-FH population.

The mechanism underlying ATX formation closely resembles that of atherosclerotic plaque formation. Given that AT structural abnormalities have been reported to occur more frequently in patients with molecularly-defined FH than in those without genetic defect [3], AT abnormalities may reflect a greater cumulative LDL-C exposure and therefore have a strong link to systemic atherosclerotic burden. The finding that AT structural abnormalities were present exclusively in patients with AT thickening suggests that such abnormalities may reflect the most advanced stage of atherosclerosis-related tendon pathology. From a clinical perspective, detection of AT structural abnormalities may allow further stratification of systemic atherosclerotic severity and thus offer additional clinical value.

Previous studies have demonstrated an association between ATT assessed by radiography and both the severity of CAD and major adverse cardiovascular events, whereas data on the association between AT structural abnormalities and systemic atherosclerosis are limited [5], [12], [13], [14], [15], [16]. Tanita et al. reported that AT structural abnormalities were identified in 21 of 353 patients undergoing PCI (6%), and that both the SYNTAX score and AT thickness were significantly higher in patients with structural abnormalities [5]. Although AT structural abnormalities were more frequently observed in the current study (14%), their associations with higher SYNTAX scores and greater ATT thickness were similarly demonstrated. Of note, in the study by Tanita et al., AT abnormalities were observed in 3.4% of patients without ATT. This likely reflects inter-observer variability, underscoring the need for well-established and objective diagnostic criteria.

Another important aspect of the present study is that the AT was evaluated using ultrasonography, which enabled assessment not only of tendon thickness but also of its internal characteristics. Traditionally, soft-tissue radiography has been commonly used for the assessment of ATT; however, its accuracy is limited by insufficient resolution to clearly delineate the boundary between the skin and the tendon. In contrast, ultrasonography provides greater accuracy and reproducibility without radiation exposure, while also allowing the visualization of internal tendon characteristics. In the current study, the presence of structural abnormalities within the AT was assessed subjectively; however, elastography represents an objective modality for evaluating the mechanical properties of the AT. Lipid deposition within the tendon has been suggested to reduce tendon stiffness, and previous studies have reported that patients with greater AT softening have a higher incidence of major adverse cardiovascular events [6], [17]. Moreover, previous studies have demonstrated that patients with FH have a lower AT elastic index than those without FH, suggesting increased tendon softness due to lipid deposition [17], [18]. Accordingly, incorporating tendon elasticity assessment in addition to AT thickness has been shown to improve the diagnostic performance of FH [18]. Further studies are therefore warranted to clarify the clinical significance of these ultrasonographic findings.

Importantly, similar associations between AT abnormalities and PVD were observed in patients without FH. Because ATX is generally considered a consequence of long-standing hyper-LDL cholesterolemia in patients with FH, a similar mechanism may apply to patients without FH. Consistent with this concept, previous studies have reported that ATX are associated with both the severity of CAD and major adverse cardiovascular events, even in cohorts that included a substantial proportion of non–FH patients [5], [12], [13], [14], [15], [16]. While the extent of AT abnormalities may differ between patients with and without FH, its pathogenesis is primarily attributed to LDL-C accumulation, suggesting that AT abnormalities may also be present in non-FH patients. The significant association between AT abnormalities and PVD observed in this cohort, which already had an elevated risk of ASCVD due to established CAD, further supports the concept that AT abnormalities may serve as an important marker of atherosclerotic burden even in non-FH patients.

## 5 Limitations

Several limitations need to be considered in our study. First, ATT has been reported to occur independently of cholesterol deposition as part of the reparative process following tendon injury and repetitive mechanical loading [2]. Therefore, the possibility that mechanical tendon stress contributed to the observed tendon thickening cannot be excluded. Data on physical activity and high mechanical stress on the AT, such as obese individuals and athletes were not available in the current study. Second, FH may have been underdiagnosed because genetic testing was not performed and untreated LDL-C levels were unavailable in some cases. In particular, patients with premature CAD may develop coronary events before manifesting clinically apparent ATX, making FH more likely to remain unrecognized in routine clinical practice. Although this may affect the results of sub-group analysis stratified by FH status, the prevalence of FH in the current study (4.2%: 14 out of 333 patients) was comparable to that reported in the previous study (3.3%), suggesting a low likelihood of underdiagnosis of FH in the current study [19]. Third, the dose and duration of prior statin therapy were unavailable, which may affect both ATT and severity of systemic atherosclerosis. Previous studies have documented the regression of AT thickness following lipid-lowering therapy in FH patients [20], [21]. Furthermore, the association between AT thickness and CAD severity has been reported to be significant only in patients without prior statin therapy, whereas this association was attenuated in those receiving statins before admission [16]. Therefore, the potential modifying effects of statin therapy should be considered when interpreting our findings. Fourth, we were unable to evaluate lipoprotein(a) and high-sensitivity C-reactive protein levels, both of which are established risk factors associated with systemic atherosclerosis. Therefore, the potential influence of these factors on the observed associations could not be assessed in the present study. Fifth, the assessment of AT structural abnormalities was based on subjective ultrasonographic interpretation. Objective measures of tendon properties, such as the elastic index, were not available in the present study. Sixth, because this was a cross-sectional study, the observed associations do not imply a causal relationship between AT abnormalities and systemic atherosclerotic burden. Longitudinal studies are warranted to determine whether AT abnormalities predict the progression of systemic atherosclerosis and future cardiovascular events.

## 6 Conclusion

AT structural abnormalities assessed by ultrasound were significantly associated with the prevalence and severity of PVD in patients undergoing PCI. Assessment of AT abnormalities may serve as a useful marker for evaluating systemic atherosclerotic burden.

The following are the supplementary data related to this article.

Supplementary tables 1-4.

## Use of AI and AI-assisted technologies statement

None.

## CRediT authorship contribution statement

**Yushi Oyama:** Writing – original draft, Methodology, Conceptualization. **Yasushi Ueki:** Writing – review & editing, Project administration. **Kyuhachi Otagiri:** Writing – review & editing, Formal analysis. **Tadashi Itagaki:** Writing – review & editing, Formal analysis. **Junko Iguchi:** Investigation. **Takuya Miyagi:** Investigation. **Takahiro Sakai:** Investigation. **Koki Fujimori:** Investigation. **Daisuke Sunohara:** Investigation. **Yuki Yamamoto:** Investigation. **Hidetomo Nomi:** Investigation. **Tamon Kato:** Investigation. **Tatsuya Saigusa:** Investigation. **Hiroshi Kitabayashi:** Investigation. **Soichiro Ebisawa:** Supervision. **Koichiro Kuwahara:** Supervision.

## Ethical approval

This study was approved by the ethics committee of Ina Central Hospital (approval no. 23–1) and was performed in conformity with the Declaration of Helsinki.

## Funding

This research did not receive any specific grant from funding agencies in the public, commercial, or not-for-profit sectors.

## Declaration of competing interest

Dr. Ueki reports grants from Astellas Pharma, personal fees from Abbott Vascular, Amgen, Bayer, Daiichi Sankyo, Kowa, NIPRO, and Novartis, outside the submitted work. Dr. Kuwahara has received lecture fees from Astellas Pharma Inc., AstraZeneca K.K., MSD K.K., Otsuka Pharmaceutical Co., Ltd., Ono Pharmaceutical Co., Ltd., Kyowa Kirin Co., Ltd., Kowa Co., Ltd., Sanofi K.K., Sumitomo Dainippon Pharma Co., Ltd. (Sumitomo Pharma Co., Ltd.), Mitsubishi Tanabe Pharma Corp., Eli Lilly Japan K.K., Nippon Boehringer Ingelheim Co., Ltd., Novartis Pharma K.K., Novo Nordisk Pharma Ltd., Bayer Yakuhin, Ltd., Pfizer Japan Inc., and Janssen Pharmaceutical K.K.; funded research or joint research expenses from Kowa Co., Ltd., AstraZeneca K.K., Daiichi Sankyo Co., Ltd., Novo Nordisk Pharma Ltd., Amgen, Janssen Pharmaceutical K.K., Parexel International Inc., and Astellas Pharma Inc. His affiliated institution (Shinshu University School of Medicine) has received grants from Otsuka Pharmaceutical Co., Ltd., Mitsubishi Tanabe Pharma Corp., Nippon Boehringer Ingelheim Co., Ltd., and Kyowa Kirin Co., Ltd., and his department has endowed chairs from Medtronic Japan Co. Ltd., Boston Scientific Japan K.K., Abbott Japan LLC, Japan Lifeline Co.,Ltd., Biotronik Japan, Terumo Corporation, Nipro Corporation, and Cordis Japan G.K.

## Data Availability

The data generated and/or analyzed during this study will be shared upon reasonable request to the corresponding author. All authors take responsibility for all aspects of the reliability and freedom from bias of the data presented and their discussed interpretation.

## References

[1] Harada-Shiba M., Arai H., Ohmura H. (2023). Guidelines for the diagnosis and treatment of adult familial hypercholesterolemia 2022. J. Atheroscler. Thromb..

[2] Kruth H.S. (1985). Lipid deposition in human tendon xanthoma. Am. J. Pathol..

[3] Junyent M., Gilabert R., Zambón D. (2005). The use of Achilles tendon sonography to distinguish familial hypercholesterolemia from other genetic dyslipidemias. Arterioscler. Thromb. Vasc. Biol..

[4] Michikura M., Ogura M., Yamamoto M. (2017). Achilles tendon ultrasonography for diagnosis of familial hypercholesterolemia among Japanese subjects. Circ. J..

[5] Tanita A., Sunamura S., Suzuki M. (2025). Structural abnormalities of the Achilles tendon are associated with coronary artery disease even without Achilles tendon thickening. Angiology.

[6] Michikura M., Ogura M., Matsuki K. (2024). Risk assessment for cardiovascular events using Achilles tendon thickness and softness and intima-media thickness in familial hypercholesterolemia. J. Atheroscler. Thromb..

[7] Mazzolai L., Teixido-Tura G., Lanzi S. (2024). 2024 ESC guidelines for the management of peripheral arterial and aortic diseases. Eur. Heart J..

[8] Matsuo H. (2009). Terminology and diagnostic criteria committee, Japan Society of Ultrasonics in medicine. Standard method for ultrasound evaluation of carotid artery lesions. J. Med. Ultrason..

[9] Kitamura A., Iso H., Imano H. (2004). Carotid intima-media thickness and plaque characteristics as a risk factor for stroke in Japanese elderly men. Stroke.

[10] Irie Y., Katakami N., Kaneto H. (2012). Maximum carotid intima-media thickness improves the prediction ability of coronary artery stenosis in type 2 diabetic patients without history of coronary artery disease. Atherosclerosis.

[11] Sianos G., Morel M.A., Kappetein A.P. (2005). The SYNTAX score: an angiographic tool grading the complexity of coronary artery disease. EuroIntervention.

[12] Kitahara H., Nakayama T., Fujimoto Y. (2020). Association between Achilles tendon xanthoma and severity of coronary artery disease in patients undergoing percutaneous coronary intervention. J. Cardiol..

[13] Fujiwara R., Yahiro R., Horio T. (2022). Achilles tendon thickness is associated with coronary lesion severity in acute coronary syndrome patients without familial hypercholesterolemia. J. Cardiol..

[14] Fujiwara R., Horio T., Yahiro R. (2023). Association of Achilles Tendon Xanthoma Identified Based on new guidelines from the Japan atherosclerosis society with coronary lesion severity in premature coronary artery disease. J. Coronary Artery Dis..

[15] Matsumoto I., Kurozumi M., Namba T. (2023). Achilles tendon thickening as a risk factor of cardiovascular events after percutaneous coronary intervention. J. Atheroscler. Thromb..

[16] Oyama Y., Ueki Y., Otagiri K. (2026). Association between Achilles tendon thickness as assessed by ultrasound and coronary artery disease severity in patients undergoing percutaneous coronary intervention. Angiology.

[17] Michikura M., Ogura M., Hori M. (2022). Association between Achilles tendon softness and atherosclerotic cardiovascular disease in patients with familial hypercholesterolemia. J. Atheroscler. Thromb..

[18] Michikura M., Ogura M., Hori M. (2021). Achilles tendon softness as a new tool for diagnosing familial hypercholesterolemia. JACC Cardiovasc. Imaging.

[19] Beheshti S.O., Madsen C.M., Varbo A. (2020). Worldwide prevalence of familial hypercholesterolemia: Meta-analyses of 11 million subjects. J. Am. Coll. Cardiol..

[20] Tada H., Okada H., Nohara A. (2022). Genetic mutations, regression of Achilles tendon thickness, and cardiovascular events among patients with familial hypercholesterolemia. Atherosclerosis.

[21] Tada H., Kojima N., Takeji Y. (2004). Impact of changes in Achilles tendon thickening on cardiovascular events in patients with familial hypercholesterolemia. Am. J. Prev. Cardiol..

