# Supplementary material for: Association between Achilles tendon abnormalities and systemic atherosclerosis in patients undergoing percutaneous coronary intervention
Source: Int J Cardiol Heart Vasc. 2026 Jul 14;65:101971. doi: 10.1016/j.ijcha.2026.101971 (PMC13382168; doi:10.1016/j.ijcha.2026.101971)
Supplement: Supplementary file 1 — Supplementary tables 1-4. [file mmc4.docx]

**Supplementary materials**

**Association between Achilles tendon abnormalities and systemic atherosclerosis in patients undergoing percutaneous coronary intervention**

Oyama Y et al.

**Supplementary Table 1.** Baseline characteristics in patients without familial hypercholesterolemia.

| Variable | ATT-/SA-  (n = 224) | ATT+/SA-  (n = 60) | ATT+/SA+  (n = 35) | *p* value |
| --- | --- | --- | --- | --- |
| Age (years) | 75 [67-81] | 76 [68-82] | 73 [65-79] | 0.325 |
| Body mass index (kg/m^2^) | 23.3 [21.6-25.3] | 22.2 [20.2-24.7] | 23.9 [22.0-26.1] | 0.037 |
| Male | 194 (87%) | 41 (68%) | 33 (94%) | 0.001 |
| Hypertension | 166 (74%) | 44 (73%) | 26 (74%) | 0.979 |
| Dyslipidemia | 168 (75%) | 41 (68%) | 25 (71%) | 0.533 |
| Diabetes mellitus | 91 (41%) | 26 (43%) | 20 (57%) | 0.190 |
| Smoking history | 159 (71%) | 33 (55%) | 24 (69%) | 0.064 |
| Family history of CAD | 31 (14%) | 7 (12%) | 2 (6%) | 0.442 |
| Renal failure (eGFR < 60 ml/min/1.73m^2^) | 111 (50%) | 39 (65%) | 25 (71%) | 0.011 |
| eGFR (mL/min/1.73m^2^) | 60 [50-73] | 55 [39-72] | 53 [30-61] | 0.008 |
| Hemodialysis | 8 (4%) | 4 (7%) | 6 (17%) | 0.008 |
| Previous PCI or CABG | 46 (21%) | 13 (22%) | 10 (29%) | 0.535 |
| Polyvascular disease | 44 (20%) | 18 (30%) | 19 (54%) | <0.001 |
| Lower extremity artery disease | 23 (10%) | 10 (17%) | 10 (29%) | 0.011 |
| ATBI or carotid artery disease | 28 (13%) | 14 (23%) | 11 (31%) | 0.007 |
| Multivessel CAD | 94 (42%) | 36 (60%) | 28 (80%) | <0.001 |
| Premature CAD | 15 (7%) | 6 (10%) | 2 (6%) | 0.614 |
| ACS presentations | 109 (49%) | 39 (65%) | 13 (37%) | 0.020 |
| Prior statin therapy | 107 (48%) | 27 (45%) | 19 (54%) | 0.685 |
| Prior ezetimibe therapy | 22 (10%) | 2 (3%) | 5 (14%) | 0.137 |
| Prior PCSK9-i therapy | 0 (0%) | 0 (0%) | 0 (0%) | N/A |
| Total cholesterol (mg/dL) | 176 [147-209] | 186 [158-204] | 168 [142-187] | 0.232 |
| LDL cholesterol (mg/dL) | 98 [77-129] | 107 [89-125] | 100 [80-119] | 0.501 |
| Triglyceride (mg/dL) | 116 [81-175] | 118 [73-164] | 103 [68-172] | 0.553 |
| HDL cholesterol (mg/dL) | 49 [41-59] | 49 [42-59] | 46 [40-53] | 0.360 |

Values are n (%) or median [interquartile range].

All continuous variables were analyzed using the nonparametric Kruskal-Wallis test.

ATT: Achilles tendon thickening, SA: Structural abnormalities, CAD: Coronary artery disease, eGFR: estimated glomerular filtration rate, PCI: Percutaneous coronary intervention, CABG: Coronary artery bypass grafting, ATBI: Atherothrombotic brain infarction, ACS: Acute coronary syndrome, PCSK9-i: Proprotein convertase subtilisin/kexin type 9 inhibitor, LDL: Low-density lipoprotein, HDL: High-density lipoprotein.

**Supplementary Table 2.** Univariate and multivariate logistic regression analysis for prevalence of polyvascular disease in patients without familial hypercholesterolemia.

| Variable | Univariate analysis | | | Multivariate analysis | | |
| --- | --- | --- | --- | --- | --- | --- |
|  | OR | 95% CI | *p* value | OR | 95% CI | *p* value |
| Achilles tendon status |  |  |  |  |  |  |
| ATT-/SA- | 1.000 (reference) |  |  | 1.000 (reference) |  |  |
| ATT+/SA- (vs. ATT-/SA-) | 1.750 | 0.922-3.340 | 0.087 | 1.990 | 1.000-3.980 | 0.050 |
| ATT+/SA+ (vs. ATT-/SA-) | 4.860 | 2.310-10.200 | <0.001 | 5.360 | 2.400-12.000 | <0.001 |
| Age | 1.030 | 1.000-1.050 | 0.039 | 1.040 | 1.010-1.070 | 0.009 |
| Male | 1.710 | 0.795-3.700 | 0.169 | 1.450 | 0.558-3.790 | 0.443 |
| Hypertension | 1.950 | 1.030-3.710 | 0.040 | 1.950 | 0.971-3.930 | 0.060 |
| Diabetes mellitus | 1.850 | 1.110-3.080 | 0.017 | 1.680 | 0.971-2.890 | 0.064 |
| Dyslipidemia | 0.965 | 0.547-1.700 | 0.903 | 0.960 | 0.514-1.800 | 0.899 |
| Smoking | 1.500 | 0.854-2.640 | 0.158 | 1.810 | 0.881-3.730 | 0.106 |
| Chronic kidney disease | 1.680 | 0.996-2.820 | 0.052 | 0.992 | 0.547-1.800 | 0.979 |

OR: Odds ratio, CI: Confidence Interval, ATT: Achilles tendon thickening, SA: Structural abnormalities.

**Supplementary Table 3.** Univariate and multivariate logistic regression analysis for prevalence of polyvascular disease in patients without hemodialysis.

| Variable | Univariate analysis | | | Multivariate analysis | | |
| --- | --- | --- | --- | --- | --- | --- |
|  | OR | 95% CI | *p* value | OR | 95% CI | *p* value |
| Achilles tendon status |  |  |  |  |  |  |
| ATT-/SA- | 1.000 (reference) |  |  | 1.000 (reference) |  |  |
| ATT+/SA- (vs. ATT-/SA-) | 1.550 | 0.798-3.020 | 0.195 | 1.630 | 0.789-3.370 | 0.187 |
| ATT+/SA+ (vs. ATT-/SA-) | 4.740 | 2.300-9.760 | <0.001 | 7.430 | 3.230-17.100 | <0.001 |
| Age | 1.030 | 1.000-1.060 | 0.023 | 1.050 | 1.010-1.080 | 0.005 |
| Male | 1.490 | 0.707-3.120 | 0.296 | 1.110 | 0.430-2.810 | 0.842 |
| Hypertension | 2.510 | 1.280-4.920 | 0.008 | 2.850 | 1.350-6.030 | 0.006 |
| Diabetes mellitus | 2.180 | 1.290-3.670 | 0.003 | 2.190 | 1.240-3.860 | 0.007 |
| Dyslipidemia | 0.945 | 0.529-1.690 | 0.848 | 0.933 | 0.485-1.800 | 0.836 |
| Smoking | 1.540 | 0.864-2.730 | 0.144 | 2.080 | 0.986-4.400 | 0.842 |
| Chronic kidney disease | 1.580 | 0.936-2.670 | 0.087 | 0.875 | 0.469-1.630 | 0.674 |

OR: Odds ratio, CI: Confidence Interval, ATT: Achilles tendon thickening, SA: Structural abnormalities.

**Supplementary Table 4.** Univariate and multivariate linear regression analysis for the SYNTAX score.

| Variable | Univariate analysis | | | Multivariate analysis | | |
| --- | --- | --- | --- | --- | --- | --- |
|  | β | t | *p* value | β | t | *p* value |
| Achilles tendon status |  |  |  |  |  |  |
| ATT+/SA- (vs. ATT-/SA-) | 0.215 | 5.968 | <0.001 | 0.293 | 5.613 | <0.001 |
| ATT+/SA+ (vs. ATT-/SA-) | 0.377 | 8.660 | <0.001 | 0.425 | 8.279 | <0.001 |
| Age | -0.030 | -0.536 | 0.592 | -0.018 | -0.322 | 0.748 |
| Male | 0.029 | 0.526 | 0.600 | 0.059 | 0.985 | 0.326 |
| Hypertension | 0.058 | 1.029 | 0.304 | 0.068 | 1.329 | 0.185 |
| Diabetes mellitus | 0.114 | 2.057 | 0.041 | 0.080 | 1.597 | 0.111 |
| Dyslipidemia | -0.044 | -0.780 | 0.436 | -0.040 | -0.799 | 0.425 |
| Chronic kidney disease | 0.163 | 2.952 | 0.003 | 0.073 | 1.360 | 0.175 |
| Smoking | -0.013 | -0.234 | 0.815 | -0.014 | -0.237 | 0.813 |

SYNTAX: SYNergy between percutaneous coronary intervention with TAXus and cardiac surgery, ATT: Achilles tendon thickening, SA: Structural abnormalities.

.

**Figure Legends**

**Supplementary Figure 1.** Study flow chart.

PCI: Percutaneous coronary intervention, ACS: Acute coronary syndrome, ATT: Achilles tendon thickening, SA: Structural abnormalities.

**Supplementary Figure 2.** Number of atherosclerotic vascular beds according to Achilles tendon status in patients without familial hypercholesterolemia.

ATT: Achilles tendon thickening, SA: Structural abnormalities.

**Supplementary Figure 3.** Association of Achilles tendon status with carotid max-IMT and SYNTAX score in patients without familial hypercholesterolemia.

1. Comparison of carotid max-IMT according to Achilles tendon status (n = 303).
2. Comparison of SYNTAX score according to Achilles tendon status (n = 308)

Max-IMT: Maximum intima-media thickness, SYNTAX: SYNergy between percutaneous coronary intervention with TAXus and cardiac surgery, ATT: Achilles tendon thickening, SA: Structural abnormalities.
